# Gaussian Mixture Models of Between-Source Variation for Likelihood Ratio Computation from Multivariate Data

**DOI:** 10.1371/journal.pone.0149958

**Published:** 2016-02-22

**Authors:** Javier Franco-Pedroso, Daniel Ramos, Joaquin Gonzalez-Rodriguez

**Affiliations:** ATVS-Biometric Recognition Group, Universidad Autonoma de Madrid, Madrid, Spain; Taxas A&M University, UNITED STATES

## Abstract

In forensic science, trace evidence found at a crime scene and on suspect has to be evaluated from the measurements performed on them, usually in the form of multivariate data (for example, several chemical compound or physical characteristics). In order to assess the strength of that evidence, the likelihood ratio framework is being increasingly adopted. Several methods have been derived in order to obtain likelihood ratios directly from univariate or multivariate data by modelling both the variation appearing between observations (or features) coming from the same source (within-source variation) and that appearing between observations coming from different sources (between-source variation). In the widely used multivariate kernel likelihood-ratio, the within-source distribution is assumed to be normally distributed and constant among different sources and the between-source variation is modelled through a kernel density function (KDF). In order to better fit the observed distribution of the between-source variation, this paper presents a different approach in which a Gaussian mixture model (GMM) is used instead of a KDF. As it will be shown, this approach provides better-calibrated likelihood ratios as measured by the log-likelihood ratio cost (*C*_*llr*_) in experiments performed on freely available forensic datasets involving different trace evidences: inks, glass fragments and car paints.

## Introduction

A likelihood ratio represents a ratio of likelihoods between two competing hypothesis. In the context of forensic science, these two hypotheses are that of the prosecution, *H*_*p*_ (for instance, the suspect originated the crime scene mark), and that of the defence, *H*_*d*_ (for instance, the suspect is not the origin of the crime scene mark). If some samples of a given material coming from a known source (*control* data) and some others coming from an unknown source (*recovered* data) are given, both known as *the evidence* (*E*), and some other information (*I*) related to the crime is available, the trier of fact (judge or jury) looks for the ratio between the probabilities of the *H*_*p*_ and *H*_*d*_ hypotheses given by

$$\begin{array}{c} \frac{P(H_{p}|E,I)}{P(H_{d}|E,I)} \end{array}$$ (1)

expressing the relative strength of one hypothesis versus the other.

However, the role of the forensic scientist must be restricted to evaluate the likelihood of the evidence assuming that any of the competing hypothesis is true, and it is not the evaluation of any other information different from that needed to evaluate the strength of the evidence. Using Bayesian theory, the above described ratio can be decomposed in the following way:

$$\begin{array}{c} \frac{P(H_{p}|E,I)}{P(H_{d}|E,I)}=\frac{P(H_{p}|I)}{P(H_{d}|I)}\cdot \frac{P(E|H_{p})}{P(E|H_{d})}=\frac{P(H_{p}|I)}{P(H_{d}|I)}\cdot LR \end{array}$$ (2)

making a clear separation of the role of the forensic scientist and that of the judge or jury. Thus, the likelihood ratio (*LR*) strengthens (*LR* > 1) or weakens (*LR* < 1) the probabilities of the propositions, in the light of the newly observed evidence. In the process of assigning/computing the *LR*, additional data, usually known in forensics as *background population*, is needed to obtain the likelihood of the parameters for the model used.

A possible statement of the hypotheses at the source level [1] is:

- *H*_*p*_: the samples found at the crime scene and those obtained from the suspect come from a common source.
- *H*_*d*_: the samples found at the crime scene and those obtained from the suspect come from different sources.

Other forms of the hypotheses are possible [1], but the analysis is outside the scope of this paper.

Likelihood ratios can be either directly derived from the data through the application of some probabilistic models (also known as feature-based LRs) or by transforming simple raw scores from a recognition system through a calibration step [2] (also known as score-based LRs). The score-based approach has been mainly used for biometric systems [3], in which the pattern recognition process does not follow a probabilistic model but a pattern matching procedure [4], the assumed conditions does not exactly hold (e.g. observations are not i.i.d. or do not follow a normal distribution), or the number of dimensions in the feature space makes the problem intractable (e.g. image vectors [5] or GMM-means supervectors [6]). However, recent approaches in face and speaker recognition modalities have begun to apply probabilistic methods with the aid of dimensionality reduction techniques [7–9]. On the other hand, the feature-based approach is usually followed in applied statistics to forensic science [10–12], where the observations are quite stable features whose within-source variation can be modelled by a normal distribution (for instance, measurements of the concentration of some chemical compounds).

A widely used approach within forensics [12–14] is that presented in [10], where the likelihood ratio is computed from multivariate data through the application of a two-level random effect model taking into account the variation *i*) between samples coming from the same source, known as *within-source* variation, and *ii*) between samples coming from different sources, known as *between-source* variation. Within-source variation is taken to be constant and normally distributed, and expressions for both normal and non-normal distribution for the between-source variation are given. When a normal distribution can not be assumed for the between-source variation, a kernel density function (KDF) [15] is used. However, as it will be shown, this KDF approach overestimates the between-source density function in some areas of the feature space for datasets where sources are grouped in several clusters.

In order to avoid this problem, an alternative approach is presented in this work, in which the between-source distribution is represented by means of a Gaussian mixture model (GMM) [16, 17], whose parameters are obtained through a maximum-likelihood (ML) criterion, with the aim of obtaining a better representation of how the parameter being modelled (sources mean) varies across the different sources observed in the background population. As being also a probabilistic method for clustering data, GMMs provide a better representation of such kind of datasets, which leads to obtain better calibrated likelihood ratios.

The rest of the paper is organized as follows. In Section [Likelihood ratio computation], the likelihood ratio computation method is presented and the generative model defined. Section [Models for between-source distribution] describes the expressions to be used for a normally distributed between-source variation and those to be used when it is represented by means of a Gaussian mixture; for this latter case, the KDF expression used in [10] is also shown. In Section [GMMs for non-normal between-source distributions], the GMM training process is described, and the differences between using the KDF and the GMM approaches are highlighted. Section [Experimental framework] describes the forensic databases, the experimental protocols and the evaluation metrics, while the results are presented and discussed in Section [Results and Discussion]. Finally, conclusions are drawn in Section [Conclusions].

## Likelihood ratio computation

In order to compute the likelihood ratio, the probability of the evidence has to be evaluated under the two competing hypothesis, *H*_*p*_ and *H*_*d*_, where the evidence consists in both the control (**y**_1_) and the recovered (**y**_2_) datasets (see the mathematical notation given in the [Appendix]). If *H*_*p*_ is assumed true, the joint probability of both datasets has to be evaluated; on the other hand, if *H*_*d*_ is assumed true, each dataset is generated from a different source and hence they are independent.

$$\begin{array}{c} LR=\frac{P(E|H_{p})}{P(E|H_{d})}=\frac{P({\text{y}}_{1},{\text{y}}_{2}|H_{p})}{P({\text{y}}_{1},{\text{y}}_{2}|H_{d})}=\frac{p({\text{y}}_{1},{\text{y}}_{2})}{p\left({\text{y}}_{1}\right)\cdot p\left({\text{y}}_{2}\right)} \end{array}$$ (3)

If a generative model with parameters Λ for the observed samples is assumed, the Bayesian solution is obtained by integrating out these parameters (if they vary from one source to another) for a given distribution which is usually obtained from a background population dataset, *p*(Λ|**X**).

$$\begin{array}{c} \frac{p({\text{y}}_{1},{\text{y}}_{2})}{p\left({\text{y}}_{1}\right)\cdot p\left({\text{y}}_{2}\right)}=\frac{\int_{\Lambda }p\left({\text{y}}_{1},{\text{y}}_{2}|\Lambda \right)\;p\left(\Lambda |X\right)\;d\Lambda }{\int_{\Lambda }p\left({\text{y}}_{1}|\Lambda \right)\;p\left({\text{y}}_{2}|\Lambda \right)\;p\left(\Lambda |X\right)\;d\Lambda } \end{array}$$ (4)

Final expressions for the numerator and denominator of the likelihood ratio will depend on the assumed generative model, which defines both the parameters Λ and the specific density functions. In this Section, we will describe the generative model used in [10], and the within-source distribution will be defined.

### The generative model

The two-level random effect model [18] used in [10] can be seen as a generative model in which a particular observed feature vector **x**_*ij*_ coming from source *i* is generated through

$$\begin{array}{c} {\text{x}}_{ij}=\theta_{i}+\psi_{j} \end{array}$$ (5)

where *θ*_*i*_ is a realization of the source random variable Θ and *ψ*_*j*_ is a realization of the additive random noise *Ψ* representing its within-source variation. This noisy term is taken to be constant among different sources and randomly distributed following

$$\begin{array}{c} \Psi ∼N(0,W) \end{array}$$ (6)

where **W** is the within-source covariance matrix. Thus, the conditional distribution of the random variable X_*i*_ (from which **x**_*ij*_ is drawn), given a particular source *i*, follows a normal distribution with mean *θ*_*i*_ and covariance matrix **W**

$$\begin{array}{c} X_{ij}|\left(\Theta =\theta_{i}\right)∼N\left(\theta_{i},W\right) \end{array}$$ (7)

Within-source covariance matrix can be computed from a background population dataset, comprising *N* = *m* ⋅ *n* samples coming from *m* different sources, through

$$\begin{array}{c} W=\frac{S_{w}}{N-m} \end{array}$$ (8)

being **S**_*w*_ the within-source scatter matrix given by

$$\begin{array}{c} S_{w}=\sum_{i=1}^{m}\sum_{j=1}^{n}\left({\text{x}}_{ij}-{\bar{\text{x}}}_{i}\right){\left({\text{x}}_{ij}-{\bar{\text{x}}}_{i}\right)}^{T} \end{array}$$ (9)

where ${\bar{\text{x}}}_{i}$ is the average of a set of *n* feature vectors from source *i*.

As the assumed generative model has only one varying parameter, *θ*, characterizing the particular source, and the observed samples are assumed i.i.d. conditioned on the knowledge of *θ*, the numerator and the denominator of the likelihood ratio given in Eq 4 can be expressed, respectively, by

$$\begin{array}{c} p\left({\text{y}}_{1},{\text{y}}_{2}\right)=\int_{\theta }p\left({\text{y}}_{1}|\theta ,W\right)\;p\left({\text{y}}_{2}|\theta ,W\right)\;p\left(\theta |X\right)\;d\theta \end{array}$$ (10)

where the parameter *θ* jointly varies for both control and recovered conditional probabilities, as they are assumed to come from the same source (say *θ*_1_ = *θ*_2_ = *θ*), and

$$\begin{array}{c} p\left({\text{y}}_{1}\right)\cdot p\left({\text{y}}_{2}\right)=\int_{\theta }p\left({\text{y}}_{1}|\theta ,W\right)\;p\left(\theta |X\right)\;d\theta \times \int_{\theta }p\left({\text{y}}_{2}|\theta ,W\right)\;p\left(\theta |X\right)\;d\theta \end{array}$$ (11)

where these conditional probabilities can be integrated out independently as they are assumed to come from different sources (say *θ*_1_ ≠ *θ*_2_).

Similarly to the random variable *X*_*ij*_, the conditional distribution of a random variable ${\bar{X}}_{i}$ representing the average of a set of *n* feature vectors {**x**_1_,**x**_2_, ‥,**x**_*n*_} coming from a particular source *i* is given by

$$\begin{array}{c} {\bar{X}}_{i}|\left(\Theta =\theta_{i}\right)∼N\left(\theta_{i},D\right),\;\;\;D=\frac{W}{n} \end{array}$$ (12)

Thus, when evaluating the conditional probability of a set of *n*_1_ control samples, **y**_1_, or a set of *n*_2_ recovered samples, **y**_2_, they will be evaluated in terms of their sample mean. That is,

$$\begin{array}{c} p\left({\text{y}}_{l}|\theta_{l},W\right)=p\left({\bar{\text{y}}}_{l}|\theta_{l},\frac{W}{n_{l}}\right)=N\left({\bar{\text{y}}}_{l};\theta_{l},D_{l}\right),\;\;\;l=1,2 \end{array}$$ (13)

This leads to the following expressions for the previously shown integrals:

$$\begin{array}{c} p\left({\text{y}}_{1},{\text{y}}_{2}\right)=\int_{\theta }N\left({\bar{\text{y}}}_{1};\theta ,D_{1}\right)\;N\left({\bar{\text{y}}}_{2};\theta ,D_{2}\right)\;p\left(\theta |X\right)\;d\theta \end{array}$$ (14)

and

$$\begin{array}{c} p\left({\text{y}}_{l}\right)=\int_{\theta }N\left({\bar{\text{y}}}_{l};\theta ,D_{l}\right)\;p\left(\theta |X\right)\;d\theta ,\;\;\;l=1,2 \end{array}$$ (15)

where only the distribution of the parameter *θ* remains undefined.

## Models for between-source distribution

Regarding the distribution *p*(*θ*|**X**) from which the parameter characterizing the source *θ* is drawn, its shape depends on how the between-source variation is modelled. In this Section, two different types of distribution of such parameter, obtained from a background population, are shown. First, we will describe the expressions for a normally distributed between-source variation. While this is not the case under analysis in this work, it will serve to derive the expressions for the non-normal case, which is expressed in terms of a weighted sum of Gaussian densities.

### Normal case

If sources means can be assumed normally distributed, Θ∼N(μ,B), then

$$\begin{array}{c} p(\theta |X)=N(\theta ;\mu ,B) \end{array}$$ (16)

where *μ* and **B** are, respectively, the mean vector and the covariance matrix of the between-source distribution. These *hyperparameters* can be obtained from a background population (with *m* sources, *n* samples per source and *N* total samples) through

$$\begin{array}{c} \mu =\frac{1}{m}\sum_{i=1}^{m}{\bar{\text{x}}}_{i} \end{array}$$ (17)

and

$$\begin{array}{c} B=\frac{S_{b}}{m-1}-\frac{S_{w}}{n(N-m)} \end{array}$$ (18)

where the between-source scatter matrix, **S**_*b*_, is given by

$$\begin{array}{c} S_{b}=\sum_{i=1}^{m}\left({\bar{\text{x}}}_{i}-\mu \right){\left({\bar{\text{x}}}_{i}-\mu \right)}^{T} \end{array}$$ (19)

Using this distribution for the parameter *θ* of the generative model, the integrals involved in the likelihood ratio computation can be written

$$\begin{array}{c} p\left({\text{y}}_{1},{\text{y}}_{2}\right)=\int_{\theta }N\left({\bar{\text{y}}}_{1};\theta ,D_{1}\right)\;N\left({\bar{\text{y}}}_{2};\theta ,D_{2}\right)\;N\left(\theta ;\mu ,B\right)\;d\theta \end{array}$$ (20)

and

$$\begin{array}{c} p\left({\text{y}}_{l}\right)=\int_{\theta }N\left({\bar{\text{y}}}_{l};\theta ,D_{l}\right)\;N\left(\theta ;\mu ,B\right)\;d\theta ,\;\;\;l=1,2 \end{array}$$ (21)

Using the Gaussian identities given in the Appendix, the numerator of the likelihood ratio can be shown to be equal to:

$$\begin{array}{c} \begin{array}{c} p\left({\text{y}}_{1},{\text{y}}_{2}\right)=N\left({\bar{\text{y}}}_{1};{\bar{\text{y}}}_{2},D_{1}+D_{2}\right)\cdot N\left({\bar{\text{y}}}^{*};\mu ,D^{*}+B\right) \end{array} \end{array}$$ (22)

where

$$\begin{array}{c} {\bar{\text{y}}}^{*}={\left(D_{1}+D_{2}\right)}^{-1}\left(D_{2}{\bar{\text{y}}}_{1}+D_{1}{\bar{\text{y}}}_{2}\right) \end{array}$$ (23)

and

$$\begin{array}{c} D^{*}=D_{1}{\left(D_{1}+D_{2}\right)}^{-1}D_{2} \end{array}$$ (24)

Finally, each of the integrals in the denominator is given by

$$\begin{array}{c} \begin{array}{c} p\left({\text{y}}_{l}\right)=N\left({\bar{\text{y}}}_{l};\mu ,D_{l}+B\right),\;\;\;l=1,2 \end{array} \end{array}$$ (25)

### Non-normal case

When the normal assumption does not hold for the distribution of sources means among the background population data, the between-source distribution *p*(*θ*|**X**) can be approximated by a weighted sum of *C* Gaussian densities in the following form:

$$\begin{array}{c} p\left(\theta |X\right)=\sum_{c=1}^{C}\pi_{c}\;N\left(\theta ;\mu_{c},\Sigma_{c}\right) \end{array}$$ (26)

where {*π*_*k*_}_*c* = 1, …, *C*_ are the weighting factors and have the following constraints

$$\begin{array}{c} 0\leq \pi_{c}\leq 1,\;\;\;\sum_{c=1}^{C}\pi_{c}=1 \end{array}$$ (27)

With this distribution as the prior probability for the parameter *θ* of the generative model, the integrals involved in the likelihood ratio computation can be written

$$\begin{array}{c} \begin{array}{cc} p({\text{y}}_{1},{\text{y}}_{2}) & =\int_{\theta }\left\{N\left({\bar{\text{y}}}_{1};\theta ,D_{1}\right)\;N\left({\bar{\text{y}}}_{2};\theta ,D_{2}\right)\;\sum_{c=1}^{C}\pi_{c}\;N\left(\theta ;\mu_{c},\Sigma_{c}\right)\right\}\;d\theta \\ & =\sum_{c=1}^{C}\pi_{c}\int_{\theta }\left\{N\left({\bar{\text{y}}}_{1};\theta ,D_{1}\right)\;N\left({\bar{\text{y}}}_{2};\theta ,D_{2}\right)\;N\left(\theta ;\mu_{c},\Sigma_{c}\right)\right\}\;d\theta \end{array} \end{array}$$ (28)

and

$$\begin{array}{c} \begin{array}{cc} p({\text{y}}_{l}) & =\int_{\theta }\left\{N\left({\bar{\text{y}}}_{l};\theta ,D_{l}\right)\;\sum_{c=1}^{C}\pi_{c}\;N\left(\theta ;\mu_{c},\Sigma_{c}\right)\right\}\;d\theta \\ & =\sum_{c=1}^{C}\pi_{c}\int_{\theta }\left\{N\left({\bar{\text{y}}}_{l};\theta ,D_{l}\right)\;N\left(\theta ;\mu_{c},\Sigma_{c}\right)\right\}\;d\theta ,\;\;\;l=1,2 \end{array} \end{array}$$ (29)

As it can be seen, the Gaussian mixture expressions become a weighted sum of the expressions given for the normal case, and so the probabilities involved in the likelihood ratio computation can be easily derived, resulting in

$$\begin{array}{c} \begin{array}{cc} p({\text{y}}_{1},{\text{y}}_{2}) & =N\left({\bar{\text{y}}}_{1};{\bar{\text{y}}}_{2},D_{1}+D_{2}\right)\cdot \sum_{c=1}^{C}\pi_{c}\;N\left({\bar{\text{y}}}^{*};\mu_{c},D^{*}+\Sigma_{c}\right) \end{array} \end{array}$$ (30)

and

$$\begin{array}{c} \begin{array}{c} p\left({\text{y}}_{l}\right)=\sum_{c=1}^{C}\pi_{c}\;N\left({\bar{\text{y}}}_{l};\mu_{c},D_{l}+\Sigma_{c}\right),\;\;\;l=1,2 \end{array} \end{array}$$ (31)

In [10], between-source distribution *p*(*θ*|**X**) is approximated through a KDF [15] where the kernel function *K*(⋅) is taken to be a multivariate normal function with smoothing parameter, or *bandwidth*, **H** = *h*^2^ **B**:

$$\begin{array}{c} p\left(\theta |X\right)=\frac{1}{{m|H|}^{1/2}}\sum_{i=1}^{m}K\left(\frac{\theta -{\bar{\text{x}}}_{i}}{H^{1/2}}\right)=\frac{1}{m}\sum_{i=1}^{m}N\left(\theta ;{\bar{\text{x}}}_{i},h^{2}B\right) \end{array}$$ (32)

where

$$\begin{array}{c} h={\left(\frac{4}{2d+1}\right)}^{\frac{1}{d+4}}m^{-1/(d+4)} \end{array}$$ (33)

As it can be seen, this Gaussian KDF is in fact a Gaussian mixture whose parameters, equating terms in Eq 26, are given by

$$\begin{array}{c} C=m,\;\;\pi_{c}=\frac{1}{m},\;\;\mu_{c}={\bar{\text{x}}}_{i},\;\;\Sigma_{c}=h^{2}B \end{array}$$ (34)

Thus, the between-source variation is approximated by an equally weighted sum of multivariate Gaussian functions placed at every source mean present in the background population, ${\bar{x}}_{i}$, being their covariance matrices given by *h*^2^ **B**. That is, a weighted version of the between-source variation is *translated* to each source mean present in the background. As we will show later on, this will lead to overestimations of the between-source density in some areas of the feature space.

## GMMs for non-normal between-source distributions

In this work, we propose to use a Gaussian Mixture Model (GMM) trained by means of a maximum-likelihood (ML) criterion in order to represent the distribution of the parameter *θ* characterizing the source. This model assumes that the observations are generated from a mixture of a finite number of Gaussian densities with unknown *hyperparameters*. Thus, it has been widely used to model the distribution of datasets in which the observations are grouped in several clusters, being each of them represented by a Gaussian density. In the case at hand, the observations are the means of the sources (${\bar{\text{x}}}_{i}$) present in the background population dataset (**X**), from which the distribution *p*(*θ*|**X**) is going to be modelled.

### GMM training

Maximum likelihood (ML) is a method of determining the parameters *Φ* of a model that makes the observed samples the most probable given that model. Conversely to KDF, where the parameters (${\bar{\text{x}}}_{i}$, **H**) are first established and the density function *p*(*θ*|**X**) arises from them, in the GMM approach the density function is obtained by maximizing the likelihood of the observed data given the model, *p*(**X**|*Φ*), from which the optimum parameters of the model are derived. In the case of a GMM of *C* components in the form of Eq 26, the ML parameters of the model, *Φ* = {*π*_*c*_, *μ*_*c*_, *Σ*_*c*_}_*c* = 1, …, *C*_, are obtained [17] by maximizing the following log-likelihood:

$$\begin{array}{c} \ln p\left(X|\Phi \right)=\sum_{i=1}^{m}\ln\left\{\sum_{c=1}^{C}\pi_{c}\;N\left({\bar{\text{x}}}_{i};\mu_{c},\Sigma_{c}\right)\right\} \end{array}$$ (35)

This can be done through the well known expectation-maximization (EM) algorithm [17, 19], which is an iterative method that successively updates the parameters *Φ* of the model until convergence. A recipe for this iterative process can be found in [17].

For a faster convergence of the algorithm, usually some steps of the *k-means* algorithm [17, 20] are previously iterated in order to obtain a good initialization of the GMM, as this clustering method provides the mean vectors {*μ*_*c*_}_*c* = 1, …, *C*_ (known as *centroids*) and the initial assignment of samples to clusters, from which {*π*_*c*_}_*c* = 1, …, *C*_ and {*Σ*_*c*_}_*c* = 1, …, *C*_ can be obtained.

The specific number of components, *C*, can be set by different methods. If the feature vectors are low-dimensional, the number of components can be visually estimated by inspecting a 2-D or 3-D projection of the background population data; however, depending on the structure of the data, there can be a lot of ambiguity in this process. Another option is to apply the *elbow method* [21] in the initial clustering stage, in which the cost function is plotted for different (increasing) number of clusters; for the first number of clusters there will be a great change when increasing the number of clusters, but at some point the marginal gain will drop indicating the proper number of clusters. A similar method can be applied by training GMMs for different numbers of components and evaluating the gain in terms of likelihood when increasing the number of them. Finally, similarly to the previous approach, if different GMMs for different number of components are trained, some model selection methods, like the Bayesian information criterion (BIC) [22] or the Akaike information criterion (AIC) [23], can be applied.

In this work, results are reported for several number of components in order to analyse how the evaluation metrics vary depending on this parameter, and the proper number of components related to the log-likelihood of the background data given the between-source density. For a given number of components, the *k-means* algorithm is iterated until convergence previously to the EM algorithm. In order to avoid local minima in *k-means* clustering, 100 random initializations are performed for a given number of components.

### GMM versus KDF

**Fig 1:**
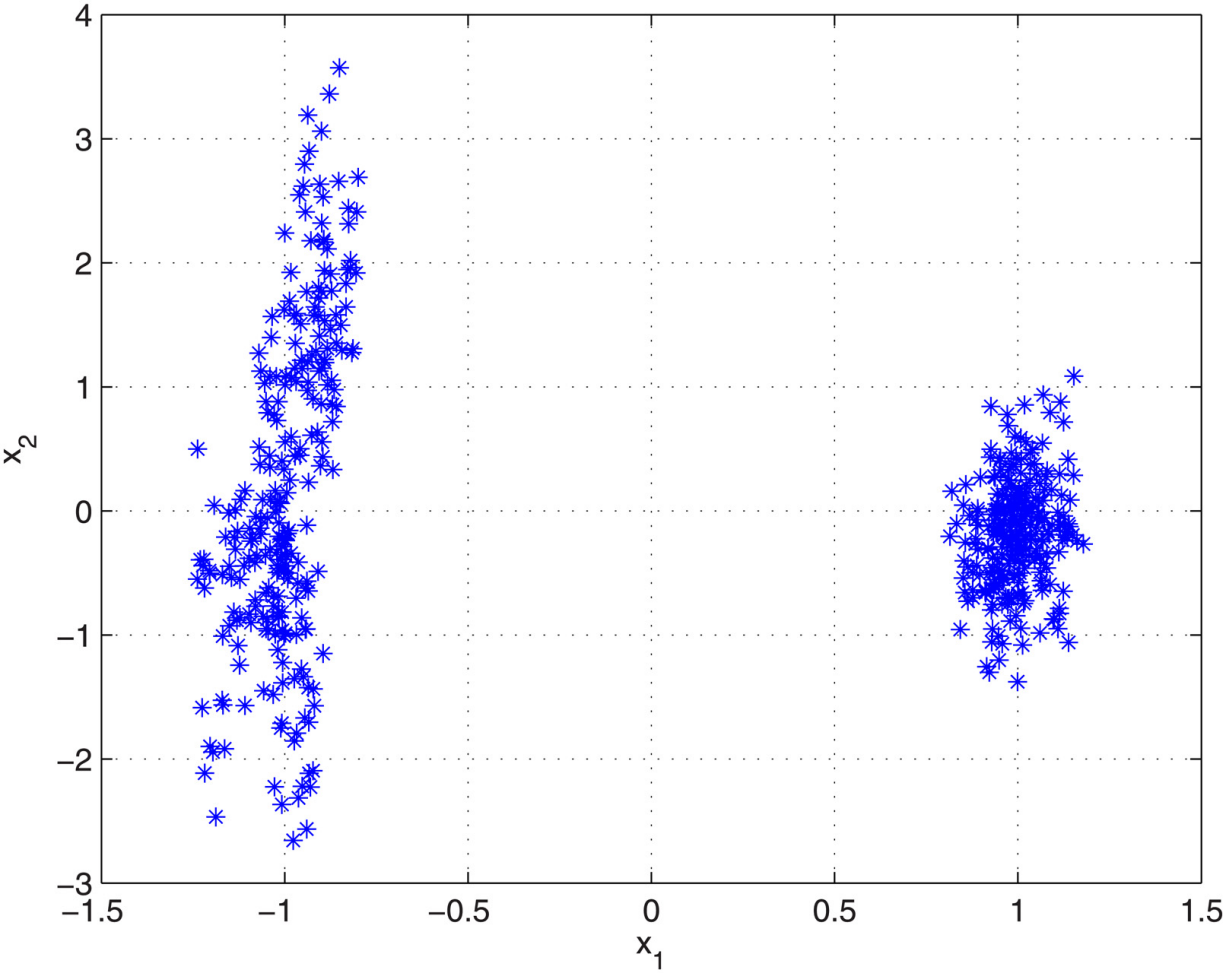
Synthetic dataset. Samples from a 2-dimensional synthetic dataset in which sources are grouped in two separate clusters.

For the purpose of illustrating the differences between KDF and GMM approaches, a synthetic 2-dimensional dataset has been generated (see Fig 1), in which 10 samples from 50 sources are drawn from normal distributions with the same covariance matrix (having then the same within-source variation). Sources means are drawn from 2 different normal distributions (25 sources each), each centred at a different separated point of the feature space, and one having a larger variance than the other in one of the dimensions. As a consequence, samples coming from different sources are grouped in two clearly separated clusters, one of them having a larger local intra-cluster between-source variation than the other. Also, the overall between-source variation is higher in one of the dimensions.

**Fig 2:**
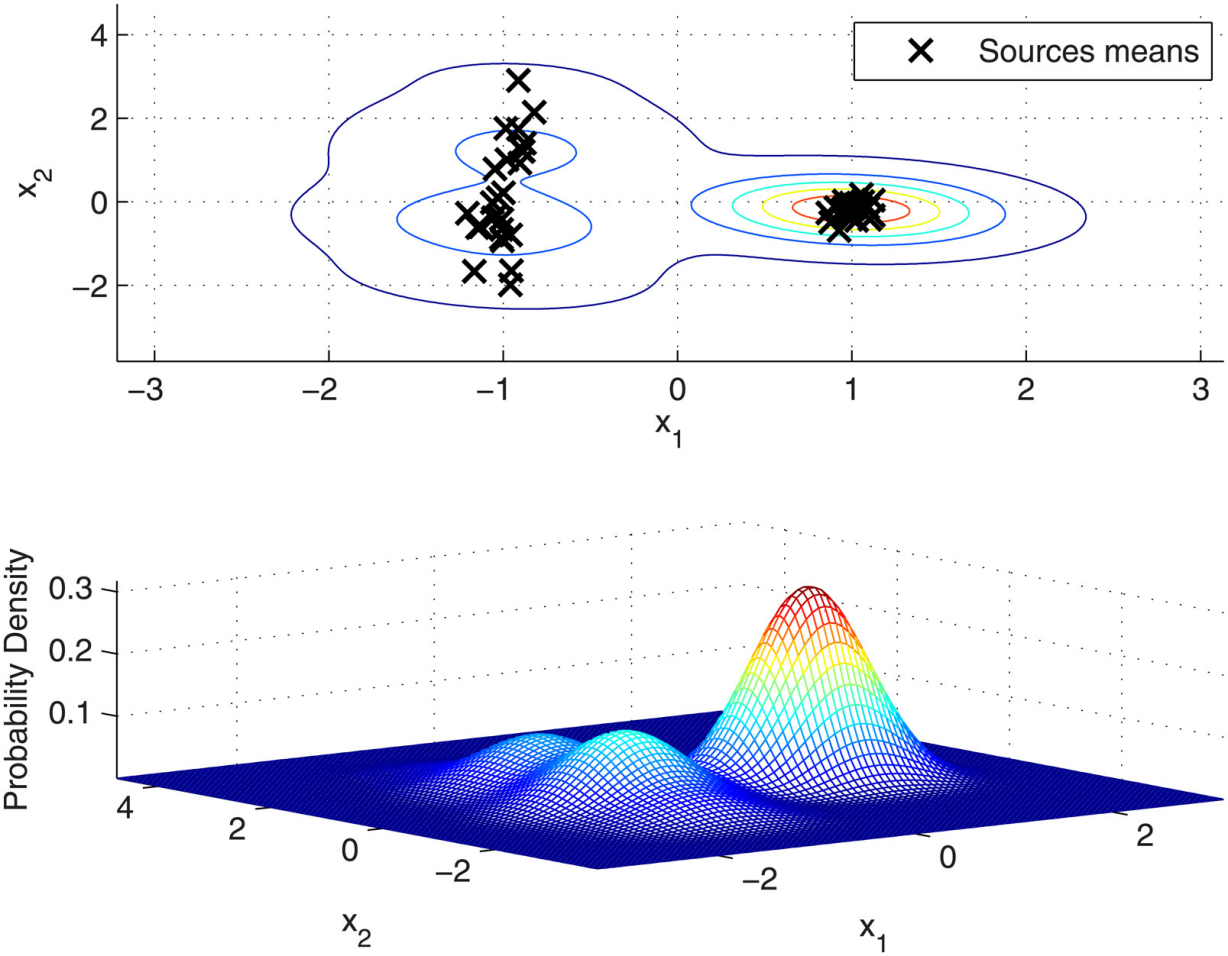
KDF modelling of between-source variation in the synthetic dataset. (Above) Sources means and level contours of the between-source density function. (Below) 3-dimensional representation of the between-source density function.

As already shown in Section [Models for between-source distribution], the density function *p*(*θ*|**X**) given by KDF approach is an equally weighted sum of Gaussian densities centred at each background source mean with covariance matrices *h*^2^ **B** (see Eq 32). Thus, a weighted version of the overall between-source variation is *translated* to every source mean, reproducing this variation locally at each source mean. The resulting density function *p*(*θ*|**X**) for our synthetic dataset can be seen in Fig 2, where it is shown that the local intra-cluster between-source variation in dimension 1 is highly overestimated for both clusters, and slightly overestimated in dimension 2 for one of them due to the larger variation in the other one.

**Fig 3:**
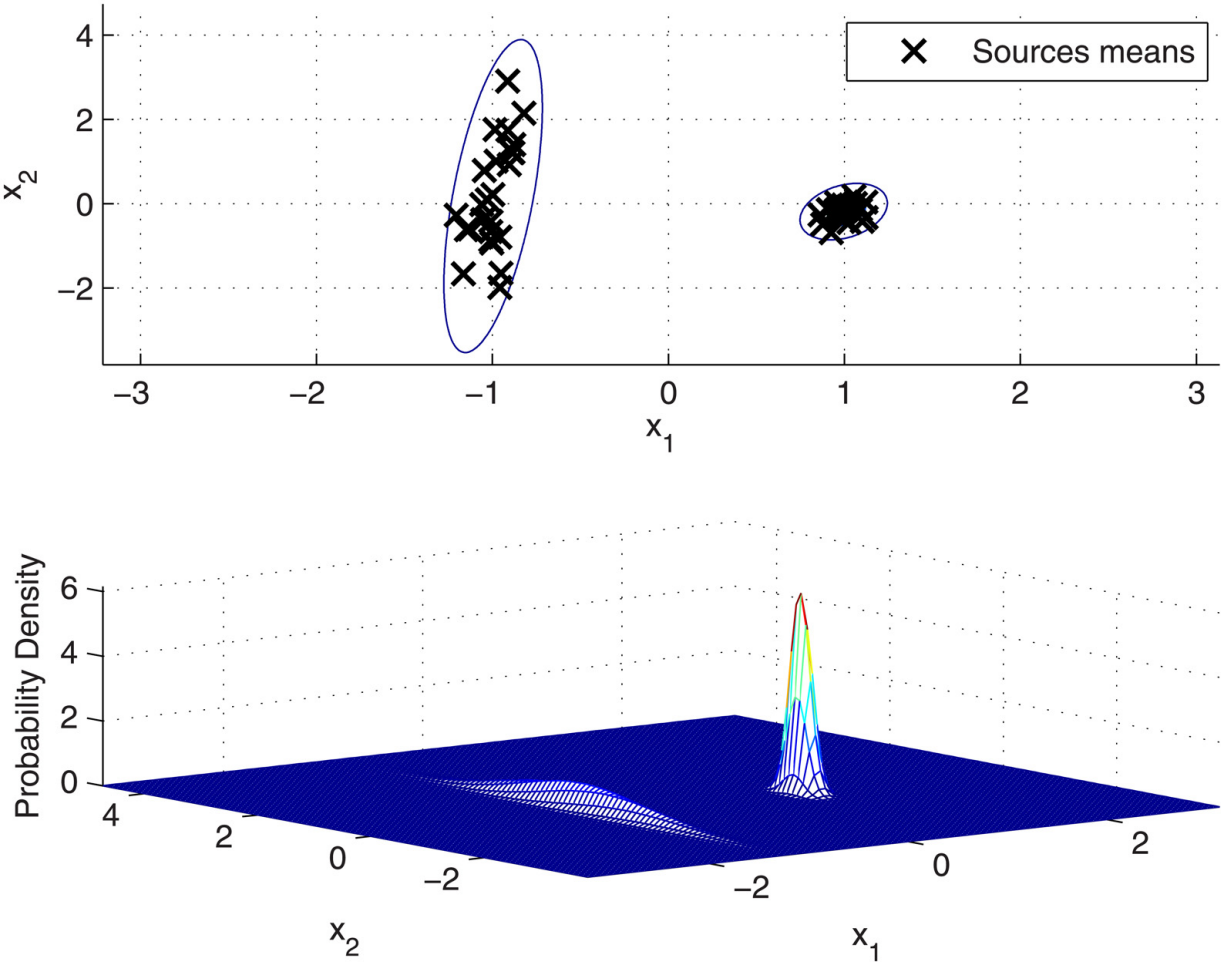
GMM modelling of between-source variation in the synthetic dataset. (Above) Sources means and level contours of the between-source density function. (Below) 3-dimensional representation of the between-source density function.

Conversely to KDF, in the GMM approach the Gaussian components are not forced to be centred at each source mean present in the background population, but a smaller number of components can be established allowing different sources means being generated from the same Gaussian component. Moreover, covariance matrices are neither fixed in advance, allowing to be locally learned for each component. As a consequence, the resulting density function can better fit the local between-source variation and the clustered nature of the dataset, as it is shown in Fig 3 for a 2-component GMM.

However, care must be taken in order to avoid *overfitting* when computing the density function through maximum likelihood. For a ML-trained GMM, the degree of fitting to the background data can be controlled through both the number of components *C* of the mixture and the number of EM iterations. In this work, for a given number of components, only two EM iterations are performed in order to avoid *overfitting*.

### Accounting for within-source variation in the background population

When training a GMM from background sources means by maximizing the log-likelihood in Eq 35, it is assumed that there is no uncertainty in these mean values. However, the number of samples per source in the background population can be limited in forensic scenarios, and so these means cannot be reliably computed. In order to account for the uncertainty in these mean values, every observation belonging to those sources can be used to train a GMM by maximizing the following log-likelihood:

$$\begin{array}{c} \ln p\left(X|\Phi \right)=\sum_{i=1}^{m}\sum_{j=1}^{n}\ln\left\{\sum_{c=1}^{C}\pi_{c}\;N\left({\text{x}}_{ij};\mu_{c},\Sigma_{c}\right)\right\} \end{array}$$ (36)

**Fig 4:**
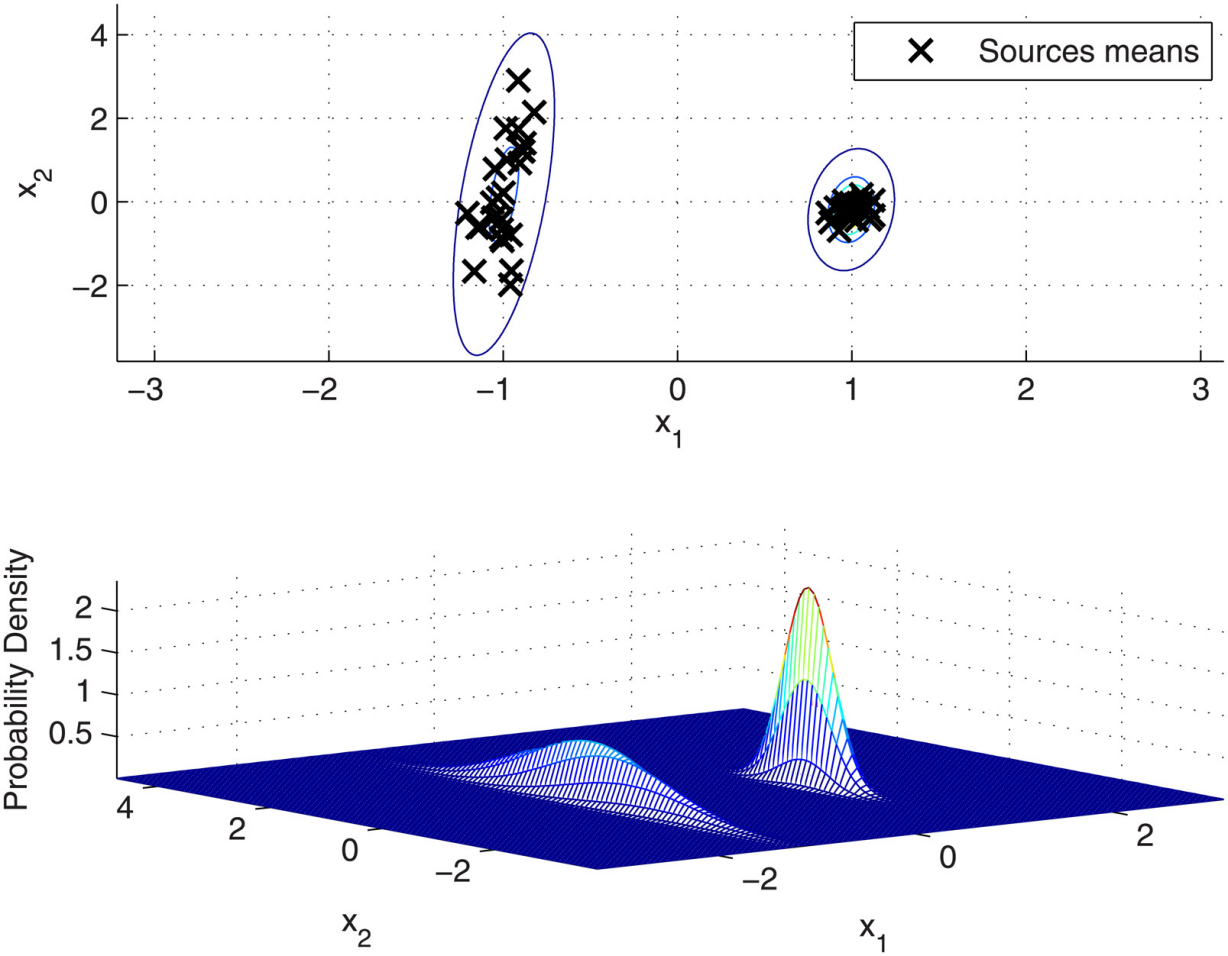
GMM modelling of between-source variation in the synthetic dataset when taking into account the background within-source variation. (Above) Sources means and level contours of the between-source density function. (Below) 3-dimensional representation of the between-source density function.

While there can be not much difference in the values obtained for components means *μ*_*c*_ in a well balanced background dataset (same number of samples per source), taking into account the variation of the samples from each source around its mean value through Eq 36 provides a more conservative background density, as every background sample is considered as a possible mean value of a source. Furthermore, this also helps to avoid Gaussian collapsing when a reduced number of sources are assigned to a particular component. The effect on our synthetic dataset is shown in Fig 4, where the Gaussian densities are placed at the same locations as in Fig 3 but larger variances and covariances are obtained, specially for the cluster with lower intra-cluster between-source variation.

## Experimental framework

### Forensic datasets

In order to test the approach proposed in this work, several types of forensic datasets have been used, being one of them the glass-fragments dataset also used in [10], which can be downloaded from http://onlinelibrary.wiley.com/journal/10.1111/(ISSN)1467-9876/homepage/glass-data.txt. A detailed description of the other two datasets can be found in [12], and can be downloaded from http://eu.wiley.com/WileyCDA/WileyTitle/productCd-0470972106.html.

- *Inks*. For this dataset, the features are the measurements of the *d* = 3 chromaticity coordinates *r*, *g* and *b* (being *r* + *g* + *b* = 1) taken on samples of blue inks. The dataset comprises the measurements on *n* = 10 samples for each of the *m* = 40 different ink sources.
- *Glass fragments*. For this dataset, the features are the measurements of the concentrations in *d* = 3 elemental ratios taken on glass fragments: log(Ca/K), log(Ca/Si) and log(Ca/Fe). The dataset comprises the measurements on *n* = 5 fragments for each of the *m* = 62 different glass sources.
- *Car paints*. For this dataset, the features are the measurements of *d* = 7 organic components present in the top layer of different acrylic car paintings. The dataset comprises the measurements on *n* = 3 samples for each of the *m* = 36 different car-paint sources.

**Fig 5:**
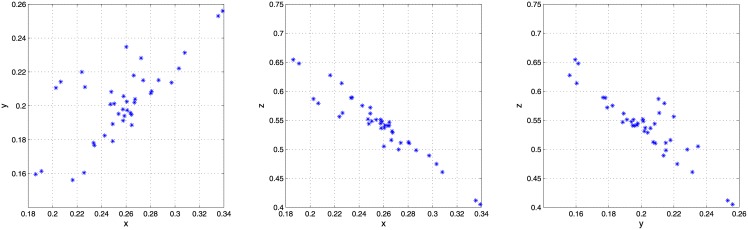
Sources means in the inks dataset. The three 2-dimensional projections of the sources means.

**Fig 6:**
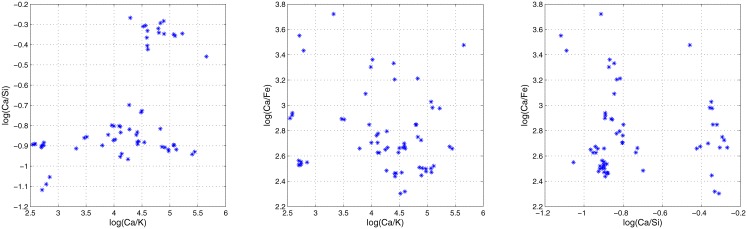
Sources means in the glass-fragments dataset. The three 2-dimensional projections of the sources means.

**Fig 7:**
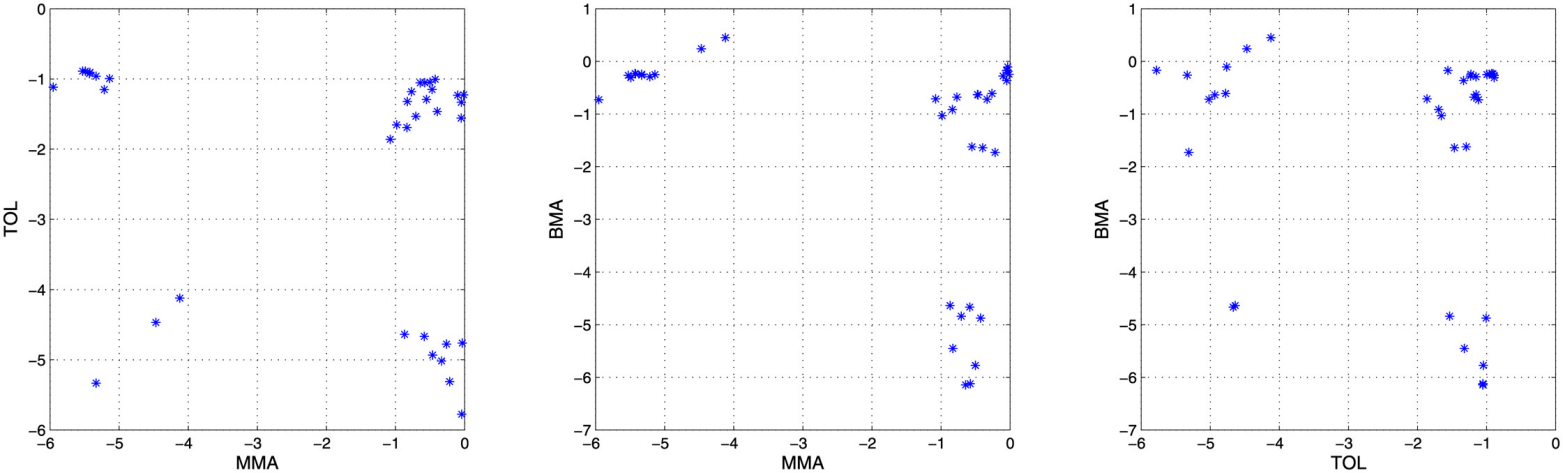
Sources means in the car-paints dataset. Three 2-dimensional projections of the sources means.

Table 1 gathers the already mentioned characteristics of these three datasets, while Figs 5, 6 and 7 show 2-dimensional projections of their sources means. As it can be seen, sources means in the last two datasets (glass fragments and car paints) present a clustered nature, while those in the first one (inks) are normally distributed [12].

**Table 1:** Datasets summary.

	m	n	d
Inks	40	10	3
Glass fragments	62	5	3
Car paints	36	3	7

m, number of sources; n, number of samples per source; d, number of features.

### Protocols

The protocol followed in [10] used the whole glass-fragment dataset in order to obtain the between-source probability density function *p*(*θ*|**X**). Then, for each source, the first 3 samples (out of 5) were used as control data and the last 3 were used as recovered data, having so both datasets one sample in common. While this *non-partitioning* protocol alleviates the lack of data due to the small size of the dataset, it may lead to overoptimistic results as the different subsets (background, control and recovered) are overlapped.

In this work, a *cross-validation* protocol is also used in order to avoid overoptimistic results, in which the dataset is divided into two non-overlapping subsets devoted to:

- obtain the between-source distribution *p*(*θ*|**X**) (*known data* or *training subset*), and
- compute same-source and different-source likelihood ratios (*unknown data* or *testing subset*). This subset is further divided into two non-overlapping halves acting as control and recovered data.

In order to alleviate the lack of data, this procedure is carried out in the following way. For each of the *m*(*m* − 1)/2 possible pairs of sources in the dataset, all the samples belonging to those two sources are taken apart from the dataset in order to be used as the *testing subset*, being the remaining sources used as the *training subset*. Each of the two sources in the *testing subset* is divided into two non-overlapping halves ({1a, 1b} and {2a, 2b}) that can be used either as control or recovered data to perform 2 same-source comparisons (1a-1b, 2a-2b) and 4 different-source comparisons (1a-2a, 1a-2b, 1b-2a, 1b-2b). Although the same control and recovered data from a particular source is used in all the different pairs in which it is involved, as the remaining sources change for each different pair, different between-source distributions *p*(*θ*|**X**) are involved in likelihood ratio computations. This procedure allow us to perform a total number of *m*(*m* − 1) same-source comparisons and 2 × *m*(*m* − 1) different-source comparisons for a given dataset, instead of the *m* same-source comparisons and *m*(*m* − 1)/2 different-source comparisons performed in [10], while the between-source distribution *p*(*θ*|**X**) used in every comparison is obtained from *m* − 2 different sources instead of *m*. The specific number of comparisons for each evaluation protocol on the different datasets are given in Table 2.

**Table 2:** Number of same-source and different-source comparisons in each dataset for the non-partitioning and cross-validation protocols.

	Non-partitioning		Cross-validation	
	Same-source	Different-source	Same-source	Different-source
Glass fragments	62	1891	3782	7564
Inks	40	780	1560	3120
Car paints	36	630	1260	2520

### Evaluation Metrics

The main evaluation metric used in order to compare the different approaches is the log-likelihood ratio cost function (*C*_*llr*_) [2, 24], which evaluates both the *discrimination* abilities of the computed log-likelihood ratios and the goodness of their *calibration*. Given a set of log-likelihood ratios $\left\{L\right\}=\left\{L_{1},L_{2},...,L_{C}\right\}$ obtained from *C* comparisons, the *C*_*llr*_ can be computed in the following way:

$$\begin{array}{c} C_{llr}\left(\left\{L\right\}\right)=\frac{1}{2\log2}\left(\frac{1}{N_{ss}}\sum_{c\in ss}\log\left(1+e^{-L_{c}}\right)+\frac{1}{N_{ds}}\sum_{c\in ds}\log\left(1+e^{L_{c}}\right)\right) \end{array}$$ (37)

where ‘*ss*’ is the set of *N*_*ss*_ same-source comparisons and ‘*ds*’ is the set of *N*_*ds*_ different-source comparisons. As it is a cost function, the larger the *C*_*llr*_ value, the worse the verification method, being *C*_*llr*_ = 0 the minimum achievable cost. Note also that this metric allows to define a *neutral reference* which does not provide support for any of the two hypothesis (that is, $L_{c}\;=0$ for every comparison), providing a reference value of *C*_*llr*_ = 1. Thus, a verification method for which *C*_*llr*_ is larger than 1 means that it is providing misleading likelihood ratios.

An important aspect of the *C*_*llr*_ is that it can be decomposed into two additive terms, one due to the discrimination abilities ($C_{llr}^{min}$) and another one due to the calibration of the verification method ($C_{llr}^{cal}$) where

$$\begin{array}{c} C_{llr}^{cal}=C_{llr}-C_{llr}^{min} \end{array}$$ (38)

and $C_{llr}^{min}$ is obtained by means of the *Pool Adjacent Violators* (PAV) algorithm [25, 26] and represents the minimum achievable *C*_*llr*_ in the case of having an optimally calibrated log-likelihood ratios set $\left\{L^{\prime }\right\}$ (details can be found in [24]).

**Fig 8:**
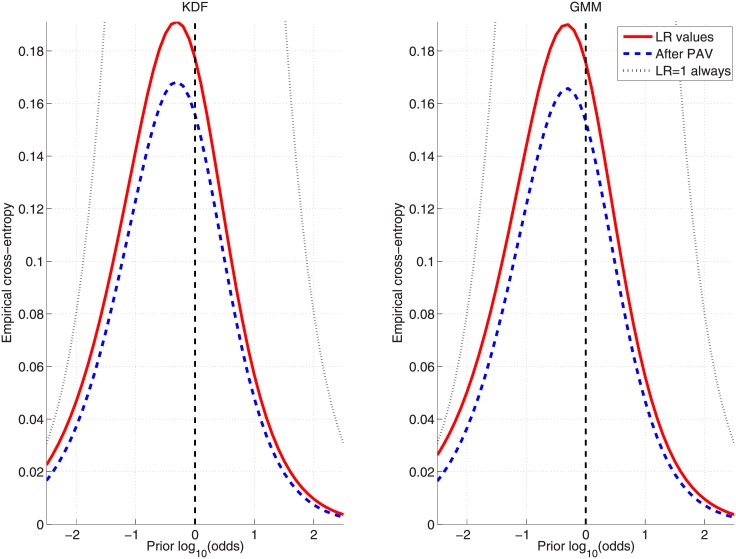
ECE plots for the KDF and GMM approaches on the inks dataset when applying the *cross-validation* protocol. GMM is trained by maximizing Eq 35.

In order to show the performance over a wide range of prior probabilities, the Empirical Croos-Entropy (ECE) plots [27, 28] will be used. These figures (see, for example, Fig 8) graphically represent what would be the accuracy (solid curve) when using the set of logLR values {L} for each of the prior probabilities (represented as logarithmic odds) in the given range. Additionally, the discriminating power is also plotted (dashed curve) for the optimally calibrated (ideal) logLRs set $\left\{L^{\prime }\right\}$, along with the neutral reference (dotted curve).

## Results and Discussion

### Inks dataset

For this dataset, as the background sources means are normally distributed, GMMs with a single component has been trained by maximizing either Eq 35 or Eq 36. Table 3 shows the detailed results (*C*_*llr*_, $C_{llr}^{min}$ and $C_{llr}^{cal}$) for KDF and GMM approaches (Eq 35 and Eq 36) when applying both the *non-partitioning* and the *cross-validation* protocols.

**Table 3:** Performance of KDF and GMM approaches on the inks dataset for the *non-partitioning* and *cross-validation* protocols.

	Non-partitioning			Cross-validation		
	$C_{llr}^{min}$	$C_{llr}^{cal}$	*C*_*llr*_	$C_{llr}^{min}$	$C_{llr}^{cal}$	*C*_*llr*_
KDF	0.1459	0.0224	0.1684	0.1558	**0.0214**	0.1778
GMM (Eq 35)	**0.1430**	**0.0223**	**0.1653**	**0.1533**	0.0223	**0.1756**
GMM (Eq 36)	0.1453	0.0271	0.1724	0.1569	0.0286	0.1855

$C_{llr}=C_{llr}^{min}+C_{llr}^{cal}$

First, it should be noted that results in the *non-partitioning* protocol are slightly better for every method as it is an overoptimistic framework where data is shared between training and testing subsets. Regarding the comparison between methods, it can be seen that no significant improvement is obtained by the GMM approach as the sources means for this dataset do not present a clustered nature. Moreover, among the two GMM variants, the results obtained when maximizing Eq 35 are slightly better, presumably due to the fact that enough number of samples per source are available (*n* = 10), compared to the number of features (*d* = 3), to compute reliable sources means, and further uncertainty accounted for Eq 36 seems to be counter-productive.

Finally, Fig 8 show ECE plots for KDF and GMM (Eq 35) approaches when applying the *cross-validation* protocol, where it can be seen that both present similar performance for a wide range of prior probabilities.

### Glass-fragments dataset

**Fig 9:**
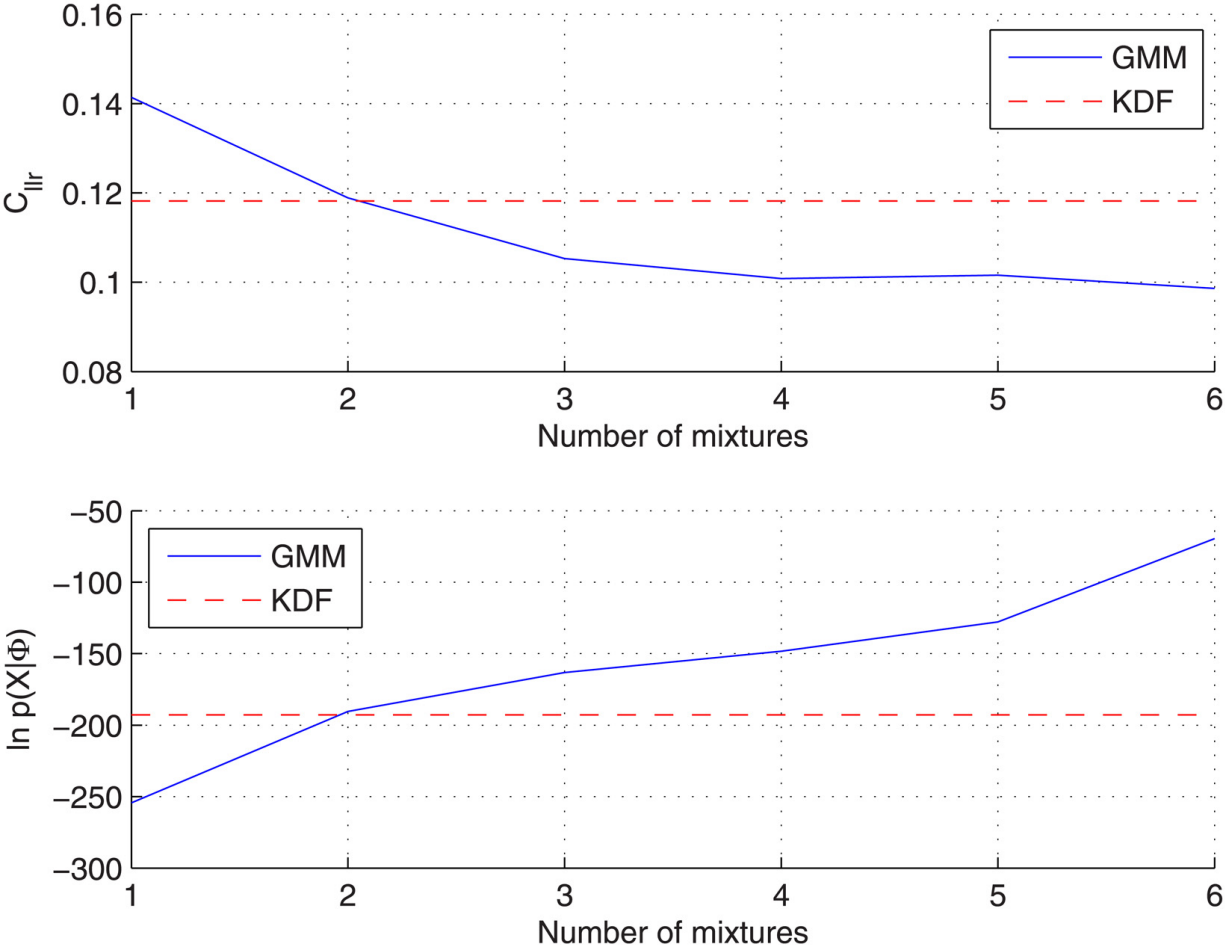
Analysis of the number of GMM components for the glass-fragments dataset when applying the *non-partitioning* protocol. GMM is trained by maximizing Eq 35. (Above) Log-likelihood ratio cost. (Below) Log-likelihood of the background data given the between-source density function.

For this dataset, several GMMs have been trained, by maximizing Eq 35, in order to analyse how the main evaluation metric (*C*_*llr*_) varies as a function of the number of components, *C*. In the experiments carried out, the maximum number of components has been limited to 6 in order to avoid Gaussian collapsing due to a reduced number of observations (sources means) per component (62 total sources in the whole dataset). Results for the *non-partitioning* protocol can be seen in Fig 9 for both KDF and GMM (Eq 35) approaches, where also the log-likelihood of the background data (sources means) given the between-source density has been plotted.

As it was expected for this *non-partitioning* protocol, *C*_*llr*_ decreases as the number of components increases, due to the shared data between training and testing subsets, which can lead to overfit the background density. However, as soon as the log-likelihood for the GMM surpass that obtained for the KDF density, better results are obtained with the GMM approach. It is also worth noting that this happens for a number of components (2–3) around that which could be expected from visual inspection of the 2-dimensional projections shown in Fig 6.

**Fig 10:**
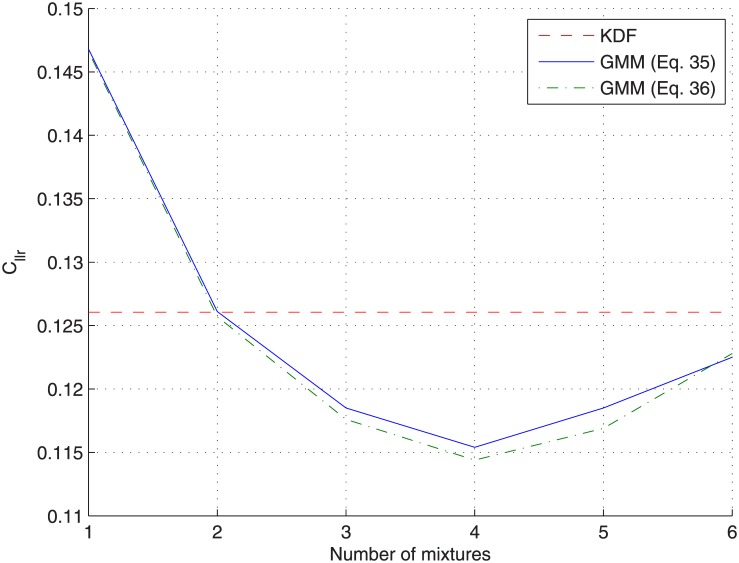
Analysis of the number of GMM components for the glass-fragments dataset when applying the *cross-validation* protocol.

Fig 10 show the same analysis for the *cross-validation* protocol. In this case, the log-likelihood is not plotted as the GMM change for every testing sources-pair (being trained on the remaining sources). Similar conclusions than before can be drawn, but here the overfitting problem affecting the *non-partitioning* protocol is revealed, as the *C*_*llr*_ for the *cross-validation* protocol reaches a minimum value for a given number of components (*C* = 4) and then increases. Results are also shown for GMMs trained by maximizing Eq 36, with similar conclusions but slightly better results, presumably due to the small number of samples per source (*n* = 5) compared to the number of features (*d* = 3).

Table 4 shows the detailed results (*C*_*llr*_, $C_{llr}^{min}$ and $C_{llr}^{cal}$) for KDF and GMM approaches (Eq 35 and Eq 36) when applying both the *non-partitioning* and the *cross-validation* protocols. For GMM approaches, results are given for the optimum number of components (*C* = 4) when the *cross-validation* protocol is applied. Again, as the *non-partitioning* protocol constitutes an over-optimistic framework, results are slightly better for every method compared to the *cross-validation* protocol. This is also the reason of obtaining better results when GMMs are trained by maximizing Eq 36, as the same sources are present in both training and testing subsets. However, when the *cross-validation* protocol is applied, there is no shared data between those subsets, and so the additional uncertainty accounted by Eq 36 provides slightly better results. In any case, both GMM approaches outperform the KDF one due to their better calibration properties for this clustered dataset.

**Table 4:** Performance of KDF and GMM approaches on the glass-fragments dataset for the *non-partitioning* and *cross-validation* protocols.

	Non-partitioning			Cross-validation		
	$C_{llr}^{min}$	$C_{llr}^{cal}$	*C*_*llr*_	$C_{llr}^{min}$	$C_{llr}^{cal}$	*C*_*llr*_
KDF	0.0787	0.0394	0.1182	0.0850	0.0410	0.1260
GMM (Eq 35)	**0.0785**	**0.0223**	**0.1008**	0.0863	0.0291	0.1154
GMM (Eq 36)	**0.0785**	0.0229	0.1013	**0.0862**	**0.0282**	**0.1144**

$C_{llr}=C_{llr}^{min}+C_{llr}^{cal}$

**Fig 11:**
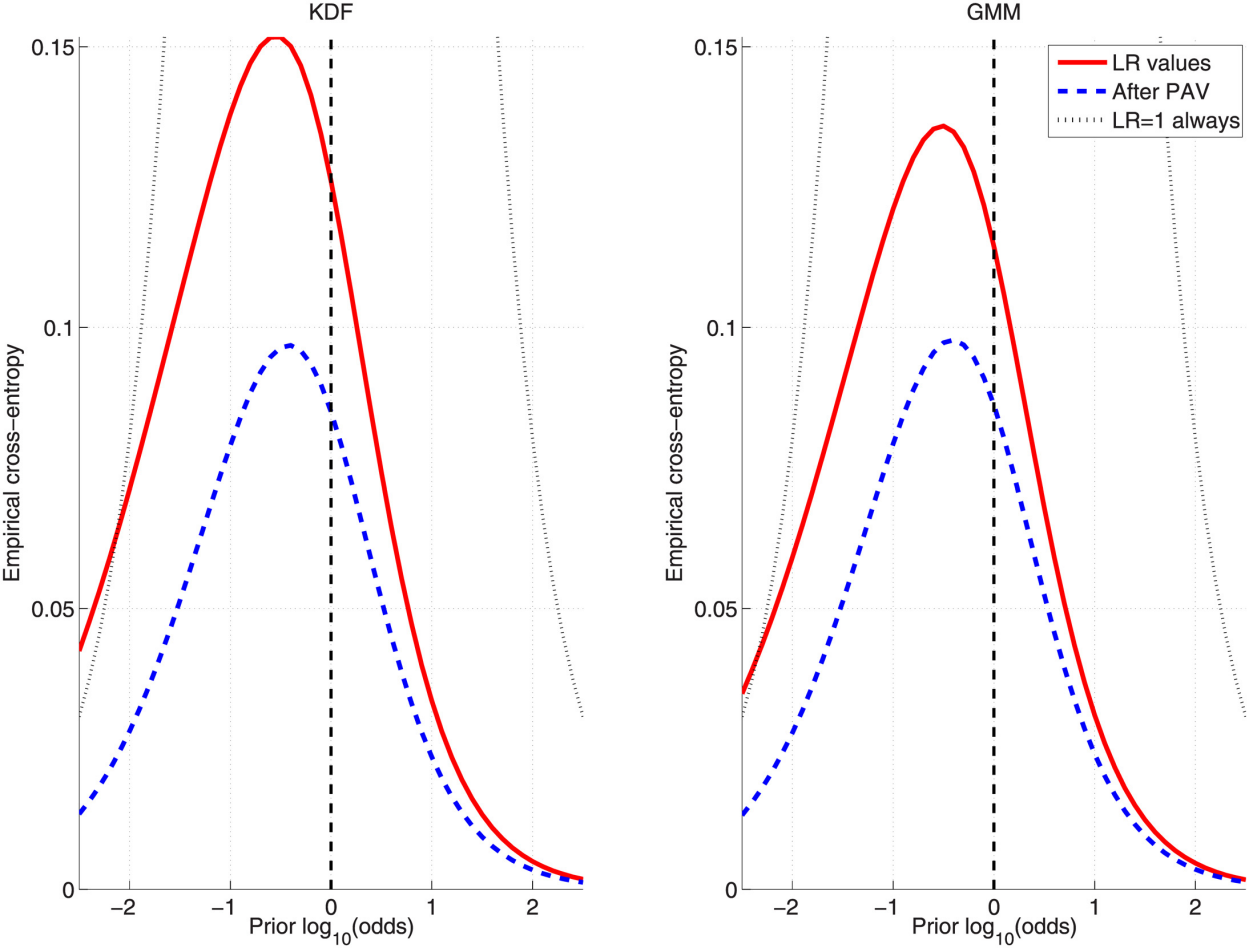
ECE plots for the KDF and GMM approaches on the glass-fragments dataset when applying the *cross-validation* protocol. GMM is trained by maximizing Eq 36.

Finally, Fig 11 shows the comparative results between KDF and GMM (Eq 36) in the form of ECE plots when the *cross-validation* protocol is applied.

### Car-paints dataset

**Fig 12:**
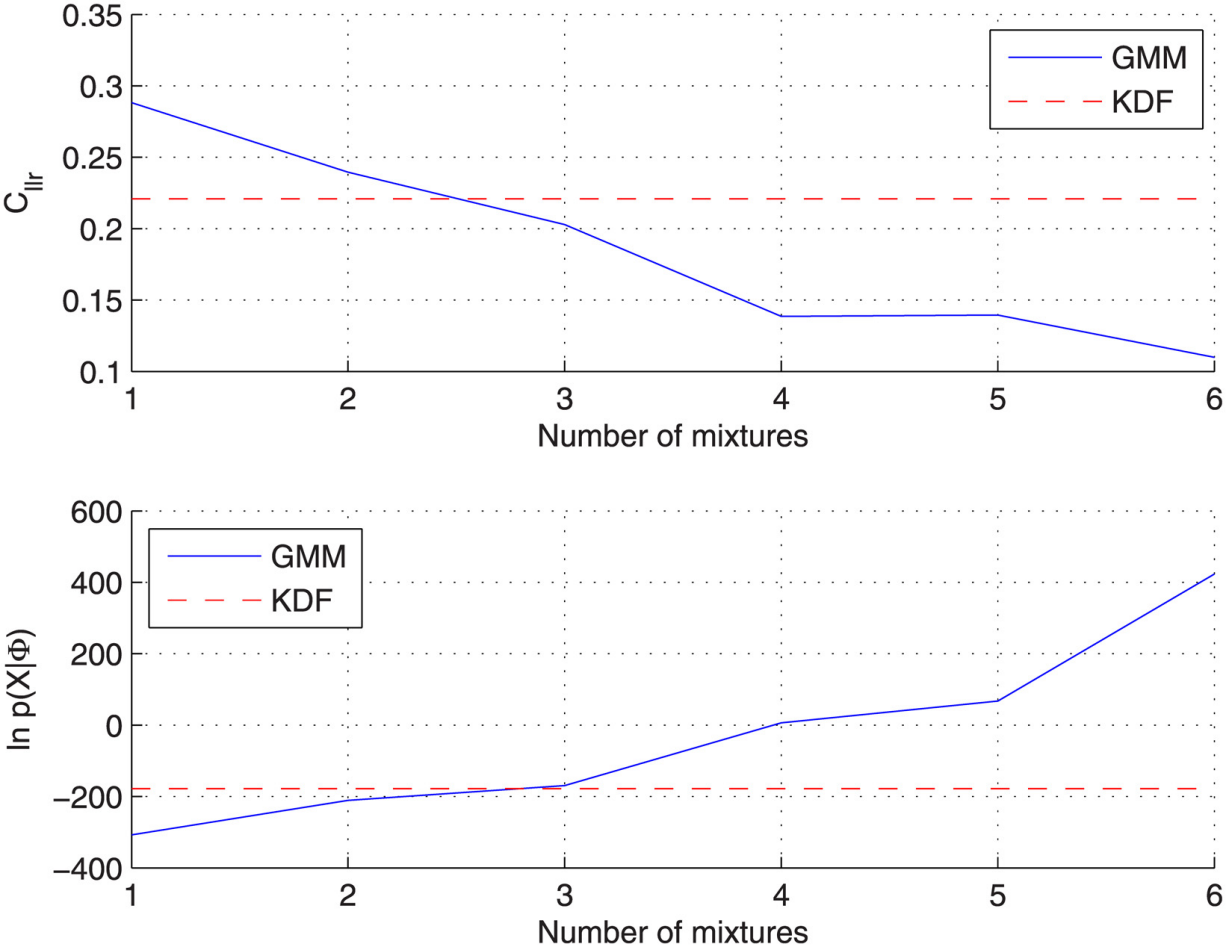
Analysis of the number of GMM components for the car-paints dataset when applying the *non-partitioning* protocol. GMM is trained by maximizing Eq 35. (Above) Log-likelihood ratio cost. (Below) Log-likelihood of the background data given the between-source density function.

An equivalent analysis to that shown for the glass-fragments dataset has been performed for the car-paints one. Fig 12 shows both the *C*_*llr*_ and the log-likelihood of the background data given the model (trained by maximizing Eq 35) as a function of the number of components for the *non-partitioning* protocol. Similarly to what happened with the previous dataset, *C*_*llr*_ decreases as the number of components increases, and as soon as the log-likelihood for the GMM surpass that obtained for the KDF density, better results are obtained with the GMM approach. Again, this happens for a number of components (3–4) around that which could be expected from visual inspection of some of the 2-dimensional projections shown in Fig 7.

**Fig 13:**
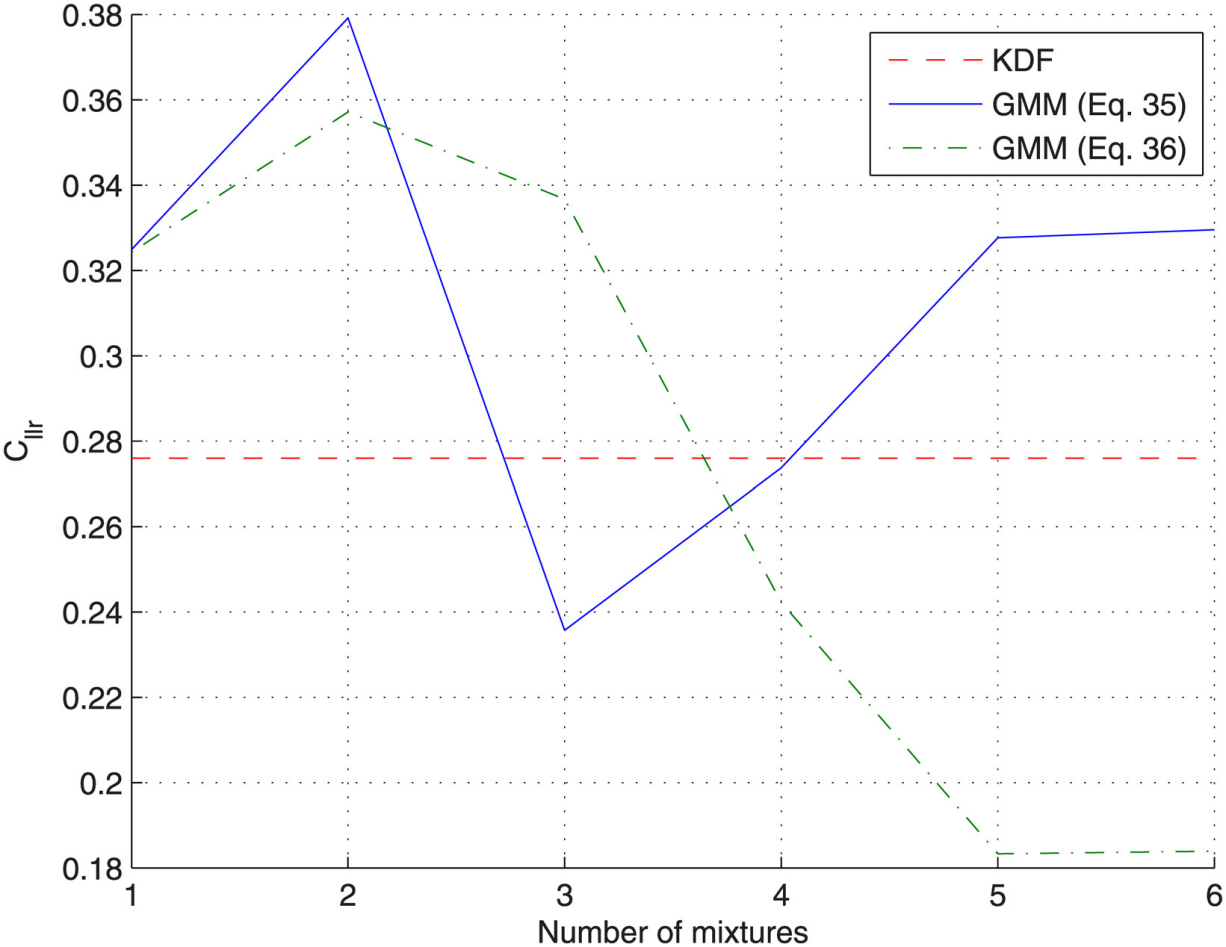
Analysis of the number of GMM components for the car-paints dataset when applying the *cross-validation* protocol.

Fig 13 show the same analysis for the *cross-validation* protocol (without showing the log-likelihood plot), where it can be seen (solid line) that, similarly to what happened with the glass-fragments dataset, a minimum *C*_*llr*_ value is reached for a particular number of components (*C* = 3) and then it increases. However, when plotting results for GMMs trained by maximizing Eq 36 instead (dot-dashed line), the number of components for which the minimum *C*_*llr*_ value is reached is slightly larger (*C* = 5); this also happens for the *non-partition* protocol, as the log-likelihood of the training data (observations) given the model for the GMM do not surpass that of the KDF until a larger number of components (*C* = 4) is reached.

Table 5 shows the detailed results (*C*_*llr*_, $C_{llr}^{min}$ and $C_{llr}^{cal}$) for KDF and GMM approaches (Eq 35 and Eq 36) when applying both the *non-partitioning* and the *cross-validation* protocols. For GMM approaches, results are given for the optimum number of components (*C* = 4 for Eq 35, *C* = 5 for Eq 36) when the *cross-validation* protocol is applied. Similar conclusions to those obtained for the glass-fragments dataset can be drawn, but much better results are obtained by GMMs approaches presumably due to the distance among clusters, which lead to KDF densities which overestimate the between-source distribution in some areas of the feature space (as shown in Fig 2 for the synthetic dataset). Among GMM approaches, the maximization of Eq 36 leads to much better results for the *cross-validation* protocol due to the small number of samples per source (*n* = 3) compared to the number of features (*d* = 7), which lead to unreliably computed sources means when training GMMs by maximizing Eq 35.

**Table 5:** Performance of KDF and GMM approaches on the car-paints dataset for the *non-partitioning* and *cross-validation* protocols.

	Non-partitioning			Cross-validation		
	$C_{llr}^{min}$	$C_{llr}^{cal}$	*C*_*llr*_	$C_{llr}^{min}$	$C_{llr}^{cal}$	*C*_*llr*_
KDF	0.0819	0.1388	0.2208	0.0972	0.1786	0.2759
GMM (Eq 35)	**0.0715**	**0.0671**	**0.1386**	0.0968	0.1769	0.2737
GMM (Eq 36)	**0.0715**	0.0729	0.1443	**0.0899**	**0.0934**	**0.1833**

$C_{llr}=C_{llr}^{min}+C_{llr}^{cal}$

**Fig 14:**
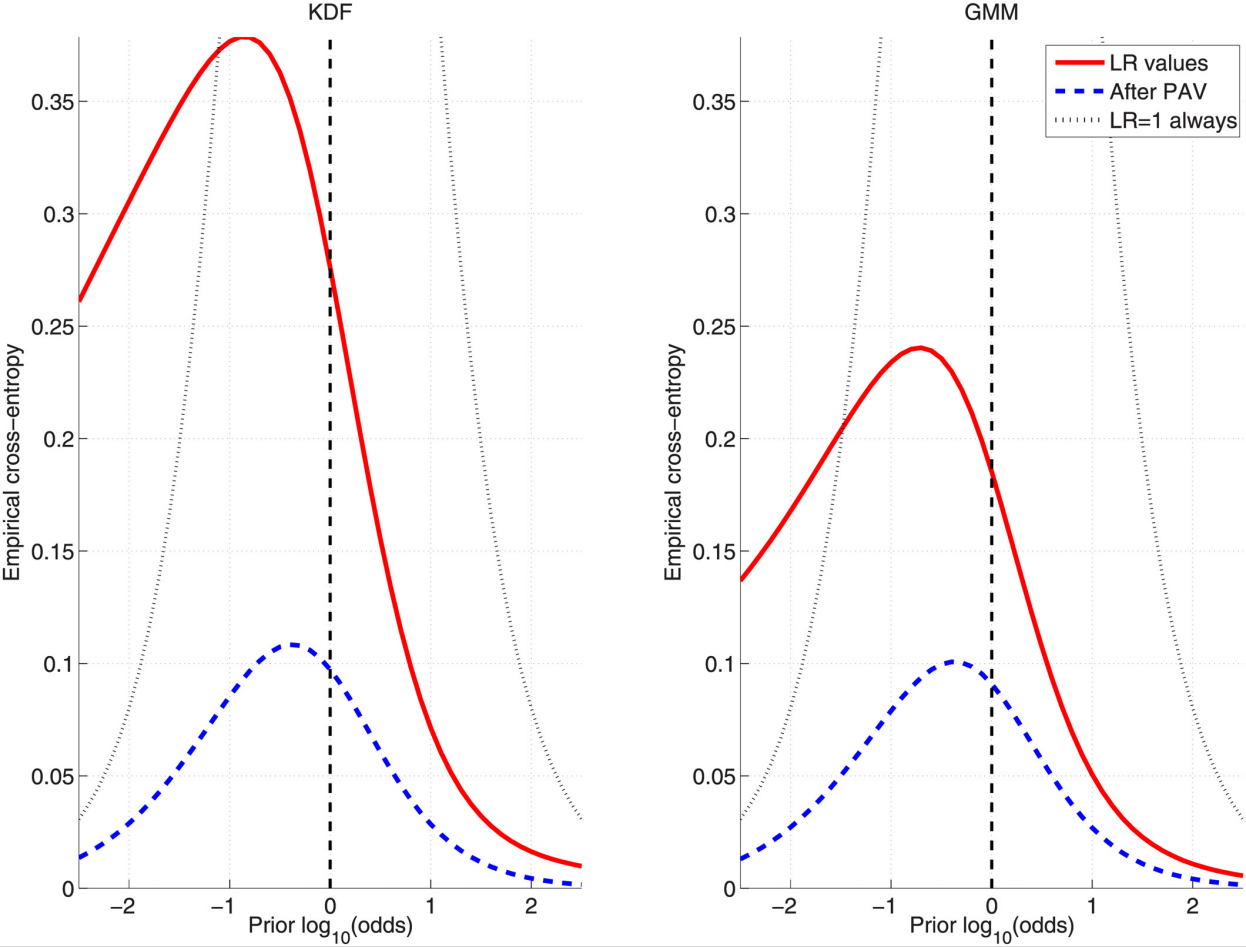
ECE plots for the KDF and GMM approaches on the car-paints dataset when applying the *cross-validation* protocol. GMM is trained by maximizing Eq 36.

Finally, Fig 14 shows the comparative results between KDF and GMM (Eq 36) in the form of ECE plots when the *cross-validation* protocol is applied.

## Conclusions

In this work, we present a new approach for computing likelihood ratios from multivariate data in which the between-source distribution is obtained through ML training of the parameters of a GMM. Using the same generative model as in [10], a common derivation of the LR expressions is presented for both Gaussian KDF and GMM, in which the between-source distribution is represented in terms of a weighted sum of Gaussian densities. Then, differences between KDF and GMM approaches are highlighted, and the effects on the obtained probability density are shown for a synthetic dataset. Furthermore, a variant in GMM training has been tested in order to account for the uncertainty in sources means when few samples per source are available in the background data.

The proposed approach has been tested on three different forensic datasets and compared with the KDF approach. Additionally to the *non-partitioning* protocol applied in [10], a more realistic *cross-validation* protocol is applied in order to avoid overoptimistic results, as ML-trained GMMs can overfit the background population density. Performance is evaluated in terms of the log-likelihood ratio cost function (*C*_*llr*_), which allows to decompose the performance in a term due to the discrimination abilities and another one due to the calibration properties. ECE plots have been used to show the behaviour in a wide range of prior probabilities, which is needed in forensic science.

Results show that, although KDF and GMM approaches present similar discrimination abilities, when the datasets have a *clustered* nature, the between-source distribution is better described by a GMM, leading to better calibrated likelihood ratios. If clusters are not easily distinguishable, the between-source distribution still can be modelled by one single component, obtaining similar results to the KDF approach. Specially remarkable are the results obtained for the car-paints dataset, where ∼50% improvement in terms of calibration performance is obtained.

## Appendix

### Mathematical notation

Throughout this work we consider multivariate data in the form of *d*-dimensional column vectors **x** = (*x*_1_, *x*_2_, …, *x*_*d*_)^*T*^. Following the same notation as in [10], a set of *n* elements of such data belonging to the same particular source *i* are denoted by **x**_*i*_ = {**x**_*ij*_}_*j* = 1, ‥, *n*_ = {**x**_*i*1_,**x**_*i*2_, …,**x**_*in*_}, while their sample mean is denoted by ${\bar{\text{x}}}_{i}$. Similarly, **x**_*i*_ is used to denote background data while **y**_*l*_ is used to denote either control (**y**_1_) or recovered data (**y**_2_). The set of feature vectors coming from different sources present in the background data is denoted by **X**.

In general, column vectors are denoted by bold lower-case letters and matrices by bold upper-case letters, while scalar quantities are denoted by lower-case italic letters. Random variables are denoted by upper-case non-italic letters. *P*(⋅) is used to indicate the probability of a certain event, while *p*(⋅) denotes a probability density function. We denote a *d*-dimensional Gaussian distribution with mean *μ* and covariance matrix *Σ* by N(μ,Σ) and the corresponding probability density function by *N*(**x**;*μ*, *Σ*) ($\text{x}\in R^{d}$).

### Multivariate Gaussian function

$$\begin{array}{c} N\left(\text{x};\mu ,\Sigma \right)={\left(2\pi \right)}^{-d/2}{\left|\Sigma \right|}^{-1/2}\exp\left\{-\frac{1}{2}{\left(\text{x}-\mu \right)}^{T}\Sigma^{-1}\left(\text{x}-\mu \right)\right\}=N\left(\mu ;\text{x},\Sigma \right) \end{array}$$ (39)

### Gaussian identities

#### Product of two multivariate Gaussian functions

$$\begin{array}{c} N(x;a,A)\cdot N(x;b,B)=N(a;b,a+B)\cdot N(x;c,C) \end{array}$$ (40)

$$\begin{array}{c} c=B{\left(A+B\right)}^{-1}a+A{\left(A+B\right)}^{-1}b \end{array}$$ (41)

$$\begin{array}{c} C=A{\left(A+B\right)}^{-1}B \end{array}$$ (42)

#### Convolution of two multivariate Gaussian functions

$$\begin{array}{c} \int_{x}N\left(x;a,A\right)\;N\left(y-x;b,B\right)\;dx=N\left(y;a+b,A+B\right) \end{array}$$ (43)

### Expressions for a normal between-source distribution

#### Derivation of the numerator

First, we solve the product of the two Gaussian functions depending on either the control or the recovered data means, obtaining the following expression

$$\begin{array}{c} \begin{array}{cc} p({\text{y}}_{1},{\text{y}}_{2}) & =\int_{\theta }\left\{N\left({\bar{\text{y}}}_{1};\theta ,D_{1}\right)\;N\left({\bar{\text{y}}}_{2};\theta ,D_{2}\right)\;N\left(\theta ;\mu ,B\right)\right\}\;d\theta \\ & =\int_{\theta }\left\{N\left(\theta ;{\bar{\text{y}}}_{1},D_{1}\right)\;N\left(\theta ;{\bar{\text{y}}}_{2},D_{2}\right)\;N\left(\theta ;\mu ,B\right)\right\}\;d\theta \\ & =\int_{\theta }\left\{N\left(\theta ;z,Z\right)\;N\left({\bar{\text{y}}}_{1};{\bar{\text{y}}}_{2},D_{1}+D_{2}\right)\;N\left(\theta ;\mu ,B\right)\right\}\;d\theta \end{array} \end{array}$$ (44)

where

$$\begin{array}{c} z={\left(D_{1}+D_{2}\right)}^{-1}\left(D_{2}{\bar{\text{y}}}_{1}+D_{1}{\bar{\text{y}}}_{2}\right) \end{array}$$ (45)

and

$$\begin{array}{c} Z=D_{1}{\left(D_{1}+D_{2}\right)}^{-1}D_{2} \end{array}$$ (46)

Being $N({\bar{\text{y}}}_{1};{\bar{\text{y}}}_{2},D_{1}+D_{2})$ independent of *θ*, we can solve the remaining integral as a convolution of two Gaussian functions:

$$\begin{array}{c} \begin{array}{cc} p({\text{y}}_{1},{\text{y}}_{2}) & =N\left({\bar{\text{y}}}_{1};{\bar{\text{y}}}_{2},D_{1}+D_{2}\right)\;\int_{\theta }\left\{N\left(z;\theta ,Z\right)\;N\left(\theta ;\mu ,B\right)\right\}\;d\theta \\ & =N\left({\bar{\text{y}}}_{1};{\bar{\text{y}}}_{2},D_{1}+D_{2}\right)\;\int_{\theta }\left\{N\left(z-\theta ;0,Z\right)\;N\left(\theta ;\mu ,B\right)\right\}\;d\theta \\ & =N\left({\bar{\text{y}}}_{1};{\bar{\text{y}}}_{2},D_{1}+D_{2}\right)\cdot N\left(z;\mu ,Z+B\right) \end{array} \end{array}$$ (47)

Finally, replacing **D**_*l*_ = **W**/*n*_*l*_, *l* = 1, 2, in **z** and **Z**

$$\begin{array}{c} z={\left(\frac{W}{n_{1}}+\frac{W}{n_{2}}\right)}^{-1}\left(\frac{W}{n_{2}}{\bar{\text{y}}}_{1}+\frac{W}{n_{1}}{\bar{\text{y}}}_{2}\right)=\frac{\frac{1}{n_{2}}{\bar{\text{y}}}_{1}+\frac{1}{n_{1}}{\bar{\text{y}}}_{2}}{\frac{1}{n_{1}}+\frac{1}{n_{2}}}=\frac{n_{1}{\bar{\text{y}}}_{1}+n_{2}{\bar{\text{y}}}_{2}}{n_{2}+n_{1}}={\bar{\text{y}}}^{*} \end{array}$$ (48)

$$\begin{array}{c} Z=\frac{W}{n_{1}}{\left(\frac{W}{n_{1}}+\frac{W}{n_{2}}\right)}^{-1}\frac{W}{n_{2}}=\frac{\frac{W}{n_{1}n_{2}}}{\frac{1}{n_{1}}+\frac{1}{n_{2}}}=\frac{W}{n_{2}+n_{1}} \end{array}$$ (49)

we obtain

$$\begin{array}{c} p\left({\text{y}}_{1},{\text{y}}_{2}\right)=N\left({\bar{\text{y}}}_{1};{\bar{\text{y}}}_{2},\frac{W}{n_{1}}+\frac{W}{n_{2}}\right)\cdot N\left({\bar{\text{y}}}^{*};\mu ,\frac{W}{n_{1}+n_{2}}+B\right) \end{array}$$ (50)

#### Derivation of the denominator

Each of the integrals in the denominator of the LR can be solved by the convolution of two Gaussian functions

$$\begin{array}{c} \begin{array}{cc} p({\text{y}}_{l}) & =\int_{\theta }\left\{N\left({\bar{\text{y}}}_{l};\theta ,D_{l}\right)\;N\left(\theta ;\mu ,B\right)\right\}\;d\theta \\ & =\int_{\theta }\left\{N\left({\bar{\text{y}}}_{l}-\theta ;0,D_{l}\right)\;N\left(\theta ;\mu ,B\right)\right\}\;d\theta \\ & =N\left({\bar{\text{y}}}_{l};\mu ,D_{l}+B\right)=N\left({\bar{\text{y}}}_{l};\mu ,\frac{W}{n_{l}}+B\right) \end{array} \end{array}$$ (51)

giving the following final expression for the denominator of the LR under the between-source normal assumption:

$$\begin{array}{c} \begin{array}{c} p\left({\text{y}}_{1}\right)\cdot P\left({\text{y}}_{2}\right)=N\left({\bar{\text{y}}}_{1};\mu ,\frac{W}{n_{1}}+B\right)\cdot N\left({\bar{\text{y}}}_{2};\mu ,\frac{W}{n_{2}}+B\right) \end{array} \end{array}$$ (52)
